# Identifying HIV-1 dual infections

**DOI:** 10.1186/1742-4690-4-67

**Published:** 2007-09-24

**Authors:** Antoinette C van der Kuyl, Marion Cornelissen

**Affiliations:** 1 Laboratory of Experimental Virology, Department of Medical Microbiology, Centre for Infection and Immunity Amsterdam (CINIMA), Academic Medical Centre of the University of Amsterdam, Meibergdreef 15, 1105 AZ Amsterdam, The Netherlands

## Abstract

Transmission of human immunodeficiency virus (HIV) is no exception to the phenomenon that a second, productive infection with another strain of the same virus is feasible. Experiments with RNA viruses have suggested that both coinfections (simultaneous infection with two strains of a virus) and superinfections (second infection after a specific immune response to the first infecting strain has developed) can result in increased fitness of the viral population. Concerns about dual infections with HIV are increasing. First, the frequent detection of superinfections seems to indicate that it will be difficult to develop a prophylactic vaccine. Second, HIV-1 superinfections have been associated with accelerated disease progression, although this is not true for all persons. In fact, superinfections have even been detected in persons controlling their HIV infections without antiretroviral therapy. Third, dual infections can give rise to recombinant viruses, which are increasingly found in the HIV-1 epidemic. Recombinants could have increased fitness over the parental strains, as in vitro models suggest, and could exhibit increased pathogenicity. Multiple drug resistant (MDR) strains could recombine to produce a pan-resistant, transmittable virus.

We will describe in this review what is presently known about super- and re-infection among ambient viral infections, as well as the first cases of HIV-1 superinfection, including HIV-1 triple infections. The clinical implications, the impact of the immune system, and the effect of anti-retroviral therapy will be covered, as will as the timing of HIV superinfection. The methods used to detect HIV-1 dual infections will be discussed in detail. To increase the likelihood of detecting a dual HIV-1 infection, pre-selection of patients can be done by serotyping, heteroduplex mobility assays (HMA), counting the degenerate base codes in the HIV-1 genotyping sequence, or surveying unexpected increases in the viral load during follow-up. The actual demonstration of dual infections involves a great deal of additional research to completely characterize the patient's viral quasispecies. The identification of a source partner would of course confirm the authenticity of the second infection.

## Review

Some confusion surrounds the earliest nomenclature of viral dual, co-, super- and re-infections, especially with regard to HIV-1. By now, it has been more or less agreed upon that viral co-infection is a double infection occurring before antibodies are detectable in the blood (before seroconversion), and that a double infection is called superinfection when the second infection takes place after seroconversion. Double infections of unknown timing are referred to as dual infections, while the term reinfection is reserved for a new infection once an initial infection has been cleared. Due to the persistence of HIV-1 infection, reinfections as defined above do not occur as the first virus is never cleared. This review will focus on double HIV-1 infections with special emphasis on superinfections, as they have attracted the most attention from an immunologic and clinical point of view.

## Super- and reinfection among different virus families

Contrary to popular belief that primary infection of an organism with a virus prevents the entry of a closely related virus, this is often not the case. In fact, the entry by the virus into the host is not prevented, but viral growth and the severity of clinical symptoms is reduced. The adaptive immune response is now primed for the incoming pathogen, commonly averting its spread and limiting subsequent damage. Thus, the strength of the response determines the precise outcome of the second infection. This principle applies both to viruses that are cleared and those that persist in the body such as retroviruses and herpesviruses. Superinfections with herpesviruses have been documented for herpes simplex virus type 1 [1], herpes simplex virus type 2 [2], Epstein-Barr virus [3], varicella-zoster virus (reviewed in [4]), cytomegalovirus [5-7] and human herpesvirus 8 [8]. Superinfections with hepatitis B virus (HBV) have also been reported, e.g. in 0.8% of chronic HBV carriers and in 1.9% of patients with acute exacerbations in Taiwan [9]. For hepatitis C virus, a virus that can be cleared, both co-, super- and reinfections have been documented (reviewed in [10]). Coinfection with retroviruses HTLV-I (human T-cell lymphotropic virus type I) and HTLV-II (human T-cell lymphotropic virus type II) has been reported in a Brazilian AIDS patient [11], but very little has been published about co- or superinfection with a same HTLV type. Dual infection with both HIV-1 and HIV-2 has already been described early in the HIV epidemic [12-14], and this finding is common in West Africa with a prevalence of 24% in HIV-infected female sex workers from Ivory Coast [15] to 40.4% in seropositive individuals from Senegal [16]. Dual infections with different strains of HIV-2 have not been described so far. In contrast, dual infections with distinct HIV-1 strains are prevalent, and form the focus of this review.

Even for viruses that persist, many uninfected cells in the host are available for infection by a second viral strain. At the cellular level, superinfection of a single cell can be prevented by a phenomenon called "superinfection resistance" (SIR). Hence, the first infecting virus actively prevents re-infection of the same cell after a short time window, usually in the range of 4–24 hrs (reviewed in [17]). The molecular mechanism of SIR has been revealed in some cases. Expression of *env *and *gag *genes in a cell interferes with subsequent viral entry of the cell and with reverse transcription of simple retroviruses such as murine leukaemia virus. The env protein is most likely involved in blocking subsequent access to receptors. More intricate systems involving accessory proteins are implicated in SIR in complex retroviruses such as feline leukaemia virus and HIV. Many retroviruses down-regulate the viral receptor on the cell surface, but this is probably not the main mechanism of SIR for HIV.

## HIV-1 superinfection: the first cases

The possibility of HIV-1 superinfection was not taken seriously for a long time, probably because the chances of acquiring a single HIV-1 infection were estimated to be low, not only for the general population but for most risk groups as well. Furthermore, it was assumed that an initial HIV-1 infection could protect against a secondary infection, as an idealized vaccine might do. Subsequently, the wealth of recombinant viruses that were detected worldwide provided the first indications that HIV-1 dual infections occur frequently, since recombinant viruses can only arise in doubly infected individuals. These dual infections were suspected to represent HIV-1 coinfections (i.e. both events occurring before HIV-1 adaptive immunity is established). However, as early as 1987, it was shown that superinfection of chimpanzees with HIV-1 by intravenous injection of a distinct strain could be achieved 6–15 months after the initial infection. Nonetheless, it was not until 2002 that the first reports of HIV-1 superinfection in humans appeared [18-20].

In three separate cases, patients were superinfected with distinct subtypes of HIV-1. In a report by Ramos et al., two intravenous drug users were superinfected with CRF01_AE (CRF = Circulating Recombinant Form) and subtype B after initial infection with subtype B and CRF01_AE, respectively [18]. In the report by Jost et al. a male having sex with men was superinfected with subtype B after a first infection with CRF01_AE [19]. In the paper by Altfeld et al., both the first and second virus were subtype B strains [20]. Thus up to 2005, 17 case reports of HIV-1 superinfection have been published (reviewed in [21,22]), and a few more cases have appeared in print since 2005 [23-29]. HIV-1 superinfection cases have also been identified in larger population studies [30-35].

## HIV-1 triple infections

To date, four patients have been described who were infected with three HIV-1 strains. Two patients were African women: a Cameroonian woman infected with a group O virus, a subtype D virus, and a subtype A/G recombinant virus [36,37]; and a patient from Tanzania infected with two subtype A strains and a subtype C virus or recombinants thereof [38]. In these women, however, it could not be established whether the triple infections were the result of coinfections or superinfections, or both. An intravenous drug user from Spain ultimately was found to carry three HIV-1 subtype B strains following a dual superinfection twelve years after primo infection [25]. Repeated superinfection was also documented for a homosexual man from the Netherlands, who was infected by a subtype B strain approximately one year after initial infection with another subtype B strain, and then with CRF01_AE one year after the second infection [24,39].

From this small number of reports, it can be concluded that infection with more than two HIV-1 strains and especially serial superinfection are rare events, which are not impossible in high risk patients. Recombinant HIV-1 strains were detected in all the above four patients, but in only one patient were viral genomes detected that mix fragments from all three strains [37]. Nevertheless, these case reports suggest that multiply infected patients could contribute to the HIV-1 viral diversity through the generation of complex recombinant viruses.

## HIV-1 superinfections and anti-retroviral therapy

Antiretroviral therapy is now commonly used in developed countries and increasingly used in the developing world. It is generally assumed, but not well established, that the incidence of HIV-1 superinfection in individuals under therapy is low, and case reports in those settings are indeed rare [27]. A productive infection will be difficult to establish as the incoming virus will immediately experience the pressure of antiretroviral drugs. No superinfections were detected during follow-up of 14 HIV-infected couples who practised high-risk behaviour, while being treated with antiretroviral drugs [40]. Despite therapy, the plasma viral load was always measurable in these patients. To facilitate detection of superinfection in this study, couples were chosen in which partners carried different HIV strains.

Some superinfections have been reported to occur during treatment interruptions [19,20] This is explained possibly because antiviral immune responses decrease during therapy. Eight superinfections have been reported which involve drug-resistant HIV-1 strains, either as the first [23,30,41] or the second [26,30] infecting virus. In some cases both viruses were found to carry (multiple) drug-resistance mutations [27,42]. One of the patients twice received a multidrug resistant (MDR) strain while not on therapy. When assessed, the replicative capacity of the drug-resistant variants was often [26,41,42], but not always [23] reduced compared to that of wild type, parental HIV-1 strains. Thus, superinfections can sometimes result in the introduction and outgrowth of a virus strain with greater fitness.

Infection with a drug-resistant virus strain severely hinders antiviral treatment options, and this is ultimately the outcome in patients infected with two MDR strains. In these cases, recombination could lead to a pan-resistant virus that cannot be treated with existing antiretroviral drugs. That such a scenario is feasible is illustrated by a case from the United States, in which a patient harbouring two MDR strains transmitted a highly drug-resistant recombinant virus [27].

## Viral sex: are HIV-1 recombinants taking over?

Recombination between HIV-1 genomes is an important viral evolutionary strategy (for reviews, see [43,44]), as it substantially enlarges the diversity of viral quasispecies within a patient [39,45]. The two copies of the RNA genome incorporated in the virus particle make HIV-1 a "diploid" virus, whereby recombinant offspring's can be produced during replication, in a manner resembling sexual reproduction. Recombinant viruses found in an epidemic can either be intra-patient [45], intra-subtype [46], or inter-subtype. In the latter two settings, infection of an initial patient with two different virus strains is a prerequisite for the formation of offspring recombinants. Inter-subtype HIV-1 recombinants, which are the most easy to identify, have been detected since the early days of the epidemic (see e.g. [47]), suggesting that multiply infected patients were present early on. For some of the strains initially classified as recombinant viruses, there has been doubt raised about their recombinant status [48], but it is obvious that many recombinant strains are circulating worldwide.

More than 20% of the current HIV-1 infections in Africa are estimated to represent recombinant strains [49]. Mathematical models indicate that a limited superinfection incidence can nevertheless lead to a high prevalence of recombinant viruses if there is a small core group of highly sexually active people and a large group of low-risk individuals [49,50]. Indeed, a higher frequency of both dual infections and recombinant strains was found in a high-risk group of bar workers in Tanzania compared to a normal-risk population of antenatal care attendees and blood donors [51]. As transmissions from these high-risk populations are likely to be frequent, it can be anticipated that HIV-1 recombinant strains will continue to expand in the HIV-1 epidemic.

This primitive sexual reproduction system might be an effective strategy for retroviruses to adapt to evolutionary constraints posed by the invasion into novel host species in the face of an error-prone viral reverse transcriptase enzyme. For vesicular stomatitis virus (VSV), an RNA virus, superinfection promotes faster adaptation than single infections [52,53]. A higher fitness of VSV populations was reached after coinfection than after superinfection, but both conditions created viruses with a higher relative fitness than those arising from single infections [53]. The authors explain this phenomenon through maximized competition for host resources between diverse populations by coinfection, whereby the fastest growing genotype from the whole genetic pool emerges, and through density-dependent selection by superinfection. Ignoring immune pressure, virus dynamics are affected in this latter model by at least three factors: the rate of exponential growth of the initial virus population, the initial decline of the population size for the secondary virus, and the finite duration of the infection passages. However, the strength of these factors reduces as the time interval between infections decreases, and adaptation is thus maximal if the time interval is zero, which equals a coinfection. Thus, in the superinfection model, the second virus swarm might contain a better competitor than any genotype present in the resident population, but the success rate of the second infecting virus is strongly context-dependent.

## Superinfection and the immune system

It is not clear whether specific host factors play a role in a productive superinfection. It has been assumed that an HIV-1 coinfection, that is a second infection before anti-HIV antibodies are detectable, can always occur, unlike a superinfection. It is likely that the adaptive immune response plays a major role in preventing a superinfection from becoming productive. It has been speculated that the lack of heterologous neutralizing antibodies predisposes the host for superinfection, as three superinfected patients showed less cross-protective and neutralizing antibody response to both autologous and heterologous HIV-1 than non-superinfected controls [54]. The authors speculated that two of their control patients with low neutralizing antibody titers should be equally susceptible to superinfection, but were less exposed. Lack of cross-neutralizing antibodies was also observed in two superinfection cases in injecting drug users from Thailand [18].

By contrast, CD8+ T-cells seem to play a less important role in protection against superinfection. A patient with strong and broadly reactive CD8+ T-cell responses that inhibit HIV-1 replication was found to be superinfected with another subtype B strain several years after the initial infection [20]. In this patient, neutralizing antibody responses to the autologous virus were weak before superinfection, as observed in other studies [18,54]; and they were not cross-reactive against the second virus. Yet, neutralizing antibody responses were measured during a period of antiretroviral treatment interruption when antiviral immunity can be expected to be low; although the CD8+ T-cell responses were powerful during that same period [20]. A later study also described a patient with an initially effective CD8+ T-cell response that successfully controlled HIV-1 replication without antiviral treatment before he became superinfected with a second subtype B strain [23]. In horses infected with equine infectious anaemia virus (EIAV, a lentivirus infecting equines), the situation seems to be the reverse. EIAV carrier horses can resist challenge with a heterologous strain in the absence of detectable cross-neutralizing antibody response to the heterologous virus [55]. Some horses immunized with an inactivated virus vaccine also resisted homologous strain challenge without detectable levels of neutralizing antibodies, but they did show virus-specific cell-mediated immune responses [56].

Thus, from the limited studies on adaptive immunity, it can be cautiously concluded that neutralizing antibody responses play a more significant role in preventing HIV-1 superinfection than CTL-responses.

## Clinical implications of HIV-1 dual infections

**Figure 1:**
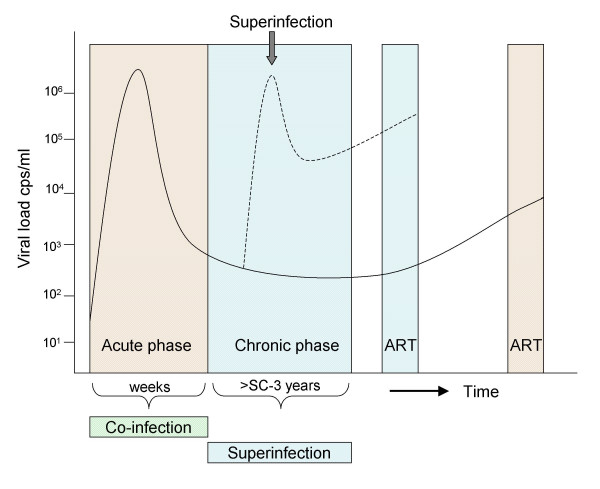
**HIV-1 plasma viral load at different clinical stages**. HIV infection is characterized by an acute phase with a high viral load, which decreases as specific immunity develops (solid line). After seroconversion (SC), the chronic phase of the infection starts, lasting several years. The chronic phase of the infection is traditionally followed by the AIDS phase, but is now increasingly replaced by the start of antiretroviral therapy (ART) in many parts of the world. An HIV-1 dual infection during the acute phase is called a co-infection, after seroconversion it is referred to as a superinfection. HIV-1 superinfections often result in an increase, sometimes temporary, of the viral load (dotted line) and an earlier start of therapy. HIV-1 superinfections in most cases are found close to the acute infection, and only rarely occur later than a few years after primary infection.

The first reports on HIV dual infections suggested an association of such findings with accelerated disease progression, particularly with clinical parameters such as a rise in the plasma viral load and a decline of the CD4+ T-cell numbers [19,57,58]. Alternatively, dual infections with fast disease progression may simply have been spotted earlier. In a doubly superinfected patient, the first superinfection was not associated with disease progression (as implied by stable CD4+ T cell counts above 500 cells/ml), while the second superinfection resulted in a permanent increase in the plasma viral load and a significant reduction in CD4+ T cells [24]. In another longitudinal study, HIV-1 superinfection was associated with rapid CD4+ T cell decline and an increased plasma viral load, necessitating the start of HAART four months later; however, in a second patient there was no decline of CD4+ T cells nor persistently increased viral load after HIV-1 superinfection ([59] and unpublished data). That some individuals are more susceptible to superinfection because they somehow lack factors to contain HIV-1 infection was hypothesized in a patient with rapid progression to AIDS [29]. This patient was superinfected with a dual-tropic (both CCR5 and CXCR4-using) HIV-1 strain 0.8–1.3 years after seroconversion that rapidly became the predominant virus strain [29]. Retrospectively, it was shown that the rapid CD4+ cell decline experienced by this patient preceded his superinfection. This suggests that fast disease progression was not completely due to a second infection with a more virulent virus, and that the already failing immune system facilitated a new HIV-1 infection. A schematic representation of HIV-1 superinfection relative to the different stages of the infection and the plasma viral load is shown in Fig. 1.

In a cohort study of African women infected with subtype C strains, dual infection was associated with an elevated viral setpoint [60]. Remarkably, two studies on HIV-1 controllers (also known as long-term non-progressors) indicated that HIV-1 dual infections are present in this patient group, but without obvious disease progression [35,61]. In one of these patients, a superinfection without any clinical deterioration occurred nine years after primo-infection, which had shown excellent immune control of the first virus [61].

Taken together, these studies suggest that HIV-1 dual infections are frequently, but not always, associated with accelerated disease progression. Due to the lack of long-term systematic investigations in a cohort setting, it is currently unclear whether HIV-1 co- and superinfections have different effects on disease development.

## Detection of dual infections

The detection and verification of HIV dual infections require extensive laboratory analyses, and it is vital that the appropriate blood samples are available. Dual infections can easily be missed, because the second infection can be transient with a very low level of virus replication [31]. There can be severe fluctuations in the relative amounts of the two viruses in subsequent plasma samples [38], which is a problem if only a single sample is analysed. Recombination can happen and the recombinant virus can outgrow parental strains, which would thus be missed [62,63]. PCR primers can be too selective, such that they do not recognize a second HIV-1 strain. It is, therefore, highly desirable that serial patient samples are available, especially from early moments, to increase the likelihood of detecting a dual infection. Very early in coinfections, we sometimes see the fast outgrowth of a single strain, with the second virus then being absent from all subsequent samples (unpublished results). One difficulty with analysis is that the second virus should not be too closely related to the first; otherwise the former will not appear as a distinct strain in a phylogenetic analysis, making it impossible to distinguish between virus evolution and superinfection. This phenomenon severely restricts the identification of novel transmissions from the same donor.

An assumed dual infection should be verified by sequencing the patient's viral quasi-species. Thus, detecting dual infections involves numerous analyses, and selecting the right group of higher-risk patients might be essential when planning large studies. Some options are available to identify patients with potential dual infections (Table 1). Serotyping based upon enzyme-linked immunosorbent assay (ELISA) which discern between HIV-1 group M (subtype B or non-B), HIV-1 group O, HIV-1 group N, and HIV-2 infections have been used to identify dual group M and O infections [36,62,64-67] and an HIV-1/HIV-2 dual infection [68]. Nonetheless, serotyping is not a means to detect HIV intra-subtype dual infections, as this method lacks discriminatory power. Caution is also warranted when using inter-subtype serotyping assays. Although specificity is generally high, discordant results have been observed [69], and not all dually reactive specimens are due to dual infection [70]. Heteroduplex mobility assay (HMA) analysis is a relatively fast and sensitive method to screen PCR amplification products for the presence of divergent sequences [34,60,71,72]. It is again important that early control samples are available. After initial selection by serotyping or HMA, PCR amplification, cloning and sequencing are necessary to confirm dual HIV infection.

**Table 1:** Methods currently used to detect HIV-1 dual infections

**Sample availability**	**Pre-selection method**	**Able to detect**	**Follow-up**	**Discovery of**	**Limitations**	**Success rate**^a^
Single sample	Serotyping (env-V3)	Different subgroups/subtypes only	Sequencing/phylogenetic analysis	Dual infection	Different subgroups/subtypes only	12.2–100%^b^
	Heteroduplex mobility assay (HMA)	Viral heterogeneity	Sequencing/phylogenetic analysis	Dual infection	Deletions in env create problems	0–19% ^c^
	Degenerate base count in RT	Viral heterogeneity	Sequencing/phylogenetic analysis	Dual infection		≥ 40% ^d^
	Multi-region hybridization assay (MHA)	Different subtypes only	None	Dual infection	Different subtypes only	Not determined
	No pre-selection	-	Sequencing/phylogenetic analysis	Dual infection	Low throughput	Low (≤ 1%)
Serial samples	Increase in viral load (VL)	-	Sequencing/phylogenetic analysis	Superinfection	Multiple factors increase VL	14–40% ^e^
	No pre-selection	-	Sequencing/phylogenetic analysis	Superinfection	Low throughput	Low (≤ 1%)

^a ^Defined as the percentage of **dual infections detected/samples pre-selected **as estimated from published studies.

^b ^HIV-1 group M/O dual infections only; Vergne et al. and Yamaguchi et al [65,66].

^c ^Manigart et al., Grobler et al., and Courgnaud et al. [34,60,72].

^d ^Cornelissen et al. [73].

^e ^Yerly et al. and Jurriaans et al. [31,59].

**Figure 2:**
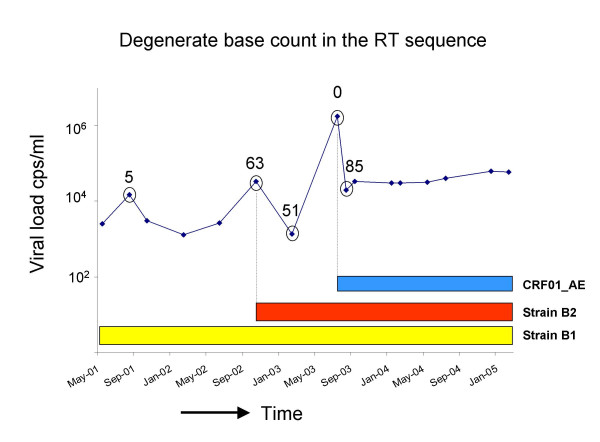
**Degenerate base counts in the RT sequence of in a triple HIV-1 infected patient**. The HIV-1 plasma viral load of an individual twice superinfected with HIV-1 is shown here to illustrate the importance of sampling time when assessing HIV-1 dual infections. The patient was infected with two different subtype B strains (indicated in yellow and red), and with CRF01_AE (blue) [24]. Degenerate base counts in the genotyping RT sequence of this patient vary from 0 at the time of the second superinfection, till 85 in the chronic phase of infection with three viral strains.

We recently described an easy method to detect dual infections that is based on the routine HIV-1 genotyping method, a population sequencing method [73]. Protease/reverse transcriptase (prot/RT) sequencing is routinely performed in the Western world to assess drug resistance mutations. If the sequences are derived from a heterogeneous population of viral DNA fragments, heterogeneous positions will show up in the sequencing electropherogram as a double or triple peak, and will be assigned a degenerate base code. Degenerate base codes are codes for incompletely specified bases in nucleic acid sequences as recommended by the Union of Pure and Applied Chemistry and the International Union of Biochemistry and Molecular Biology (IUPAC-IUBMB) that signify double (R = A or G; Y = C or T; K = G or T; M = A or C; S = G or C; W = A or T), triple (e.g. B = C, G, or T; D = A, C, or T; H = A, C, or T; V = A, C, or G) or quadruple (N = A, C, T, or G) bases in a DNA sequence [74]. From the polymerase gene sequences that yielded a high score of degenerate base codes, we measured a high percentage of dual infections. If the number of degenerate base codes in the reverse transcriptase (RT) fragment of the polymerase sequence is 34 or more, 43% of patients were confirmed to be dually infected [73]. This percentage rose to 73% when degenerate base codes in RT increased to 45 or more. In the other patients, heterogeneity could be ascribed to massive viral evolution. Figure 2 shows the variation of degenerate base counts in RT over time in a patient twice superinfected with HIV-1. From this example it is apparent that a superinfection can easily be missed when testing only a single sample, as during the acute phase of the second superinfection when most of the viral RNA originated from the incoming virus, and thus no heterogeneity was detected in the RT sequence.

To detect HIV-1 superinfections, one should also be aware of sudden, unexpected rises in the viral load of at least 10-fold [31,59]. HIV-1 superinfection is frequently accompanied by a steep rise in the plasma viral load. The first HIV-1 superinfections in patients were identified mainly because of such unexplained viral load increases [19,20,22,24]. In 5 chronically infected intravenous drug users with an unexpected rise in the viraemia, 2 experienced a superinfection with a different HIV-1 subtype [31]. A study of untreated patients experiencing a sudden rise in the plasma viral load indicated a superinfection in 2 out of 14 patients [59].

The only method currently available to detect some dual infections without further sequence analysis is the Multi-region Hybridization Assay (MHA), which is based on real-time PCR amplification of multiple genomic fragments, using and subtype-specific probes for detection. These MHA's can only be used in areas were multiple subtypes prevail, such as in Africa [75] or Asia [76], and will obviously miss intrasubtype dual infections. The success rate of the different pre-selection methods as calculated from published studies is variable (Table 1). Overall, the number of degenerate bases in RT is the best predictor of an HIV-1 dual infection, followed by HMA analysis. Dual reactivity in serological assays (serotyping) is highly predictable of intergroup dual HIV-1 infections, but intragroup predictability is limited [70]. Using no pre-selection method results in very low success rates, as dual infections are relatively uncommon in all cohorts examined.

No matter what method was used to pre-select a suspected HIV dual infection, actual confirmation requires a phylogenetic analysis of viral sequences. To construct such a phylogenetic tree, several methods are available (see e.g. [77]). The neighbour-joining (NJ) method and, more recently, Bayesian inference of phylogeny, an approach similar to maximum-likelihood, are often used in HIV evolution studies [78]. Whatever the method used to construct the tree, it is important that some statistical significance is given to the branching of sequences. With the NJ method, this is commonly done by applying a bootstrapping algorithm to the tree. For Bayesian trees, posterior probabilities can be calculated. Bootstrap numbers of 80 or over and posterior probabilities of 0.8–1.0 are generally taken as positive evidence for the accuracy of a cluster of sequences.

We outlined earlier [73] a number of criteria for the positive identification of HIV-1 dual infection based upon sequence analysis that should be true with two distinct phylogenetic methods: 1. Sequences of a single patient should cluster independently, or 2. Sequences of a patient cluster together, but the bootstrap/posterior probability value connecting the branches should be low (values under 80/0.8 are normally considered insignificant). Divergent sequence groups from a patient that cluster together with high confidence levels should always be attributed to viral evolution and not to dual infection. For definite proof of HIV superinfection, some scientists consider it essential to couple the sequences from a suspected superinfection case to those of an identified source partner. This can also be done by phylogenetic analysis of viral sequences. However, in many cases, especially with anonymous sexual contacts, the source will not be easy to identify. Sometimes, an indication of transmission may be retrieved from sequence databases [27,79-82].

## Timing of HIV superinfection

Superinfections have been described after long-term chronic infection [25], but the most optimal period for a second infection seems restricted to a window period of less than 3 years after the initial infection (see also [49]). The first few months after primary infection appear therefore to be the most favourable for superinfection. Such a window was described for macaques infected with simian immunodeficiency virus or HIV-2, although in monkeys the time of susceptibility to a second infection appears to be even shorter; no more than a few weeks after the first infection [83-88]. This uneven distribution of susceptibility to HIV superinfection suggests that the immune system is an important player in the defence against superinfection. As the adaptive immune response is usually in place within a few weeks to months after initial infection, it can be reasoned that in some patients either an effective immune response takes longer to mature, or that the immune system is quickly deteriorating, allowing a superinfection, or that other factors play a decisive role. Cellular and viral kinetics are also important for the susceptibility to and the timing of HIV-1 superinfection.

From studies of macaques infected with SIV it became clear that a main target site of SIV infection is the gut-associated lymphoid tissue [89]. At the acute stage, a massive infection and subsequent destruction of 60–80% of memory CD4+ T cells takes place in the mucosa and lymph nodes, with initially little effect upon the peripheral blood CD4+ T cell numbers, mainly because local memory CD4+ T cell numbers are low [89-91]. Subsequently, CD4+ T cells from other compartments travel to the mucosa to replenish the lost T cells, and peripheral blood CD4+ T cells slowly start to decline. These findings were confirmed in HIV-1 infected humans [92]. Initiation of HAART early in HIV infection resulted in a near-complete restoration of intestinal CD4+ T cells, but this was not the case if HAART is started during chronic infection. So how can HIV-1 superinfection occur when its main target cells are largely gone, and unlikely to return? Several factors could play a role. In macaques, the relative virulence of the infecting strain was associated with the rapidity and degree of T cell depletion in the intestine [89]. Infecting monkeys with the SIVmac239Δ*nef *straindid not result in a significant depletion of intestinal CD4+ T cells [89]. In humans, long-term nonprogressors were found to maintain normal CD4+ T cell numbers not only in peripheral blood, but also in the intestinal mucosa [93]. This suggests that both viral and host factors determine the extent of initial memory CD4+ T cell depletion in the host, and thus the susceptibility to HIV-1 superinfection. Sufficient memory CD4+ T cells to support a second HIV-1 infection should thus remain in patients infected with a less virulent virus, in patients efficiently controlling their HIV-1 infection (long-term nonprogressors), and in individuals who have started HAART very early in infection. Besides, it is possible that the 20–40% of memory CD4+ T cells that are left uninfected in the mucosal tissues of the average patient is enough to support a second HIV-1 infection, especially if new CD4+ T cells are recruited to these tissues.

## Are HIV-1 superinfections increasing?

A major risk factor to acquire a second HIV infection is risk exposure, which itself consists of two aspects: risky behaviour and HIV-1 prevalence. Decreasing risky behaviour and the absence of HIV infected individuals surrounding the patient will diminish the frequency of HIV superinfections. If the HIV infection rate is relatively high, such as in areas where the epidemic is well established, then HIV dual infection rate will be higher than in regions where HIV was introduced more recently. This is reflected in studies in sub-Saharan Africa where HIV is highly prevalent in most populations, with many people demonstrating high risk behaviour. A study published in 2004 on HIV-1 dual infection in a cohort of commercial sex workers in Burkina Faso found that 2 out of 147 women were dually infected [34]. A similar study in South Africa, published in 2004, showed that within 3 months of infection, 19% (6 out of 31) of female sex workers were dually infected with distinct subtype C viruses [60]. Among female bar workers in Kenya, who are less exposed than sex workers, three cases of HIV-1 superinfection were detected in 20 persons examined (15%), suggesting that HIV-1 superinfection occurs as commonly as primary infections [33]. In a similar risk group of bar workers in Tanzania, 19% of dual HIV-1 infections were seen, compared with 9% in the normal risk population of antenatal care attendees and blood donors [51]. These figures correlate well with the HIV-1 prevalence data from the countries involved [94]. HIV prevalence is the lowest in Burkina Faso (4.2% among the adult population in 2004, of which 88% HIV-1 and 12% HIV-2), intermediate in Kenya (7.4% among adults in 2004) and Tanzania (6.4–11.9% in 2003), and highest in South Africa with an estimated prevalence of 17.8–24.3% among the adult population in 2003.

Apart from heterosexual contacts, HIV-1 can be transmitted in several other ways. Among intravenous drug users (IDUs), the virus can be injected directly into the blood stream by means of used needles, providing an easy opportunity for infection as no mucosal barriers need to be taken. One would expect that the likelihood of productive superinfection is high in this risk group. In one cohort study of IDUs in Switzerland, 3 coinfections were found among 58 seroconverters [31]. At a later time point, 1 of 40 (2.5%) of these seroconverters superinfected. In a similar study in Thailand, no dual infection was seen among 126 seroconverting IDUs [32]. During follow-up a year later, 2 of 80 (2.5%) IDUs had acquired an HIV-1 superinfection, a number that is very similar to that of the Swiss cohort. In contrast, none of 37 IDUs with high-risk behaviour was superinfected during the 1987–2000 period in the San Francisco Bay area [95]. Similarly, no HIV-1 superinfections were detected in 9 Brazilian IDUs with continuing high-risk practices [96]. These numbers indicate a similar rate of HIV-1 dual infections in IDU cohorts when compared to African heterosexual cohorts. The absence of superinfections in the latter two groups may be due to a low incidence or the availability of effective therapy measures, although use of HAART was infrequent in the Brazilian study. A mathematical analysis suggested that 9% of new infections among IDU in Thailand represent superinfections [97].

No systematic studies on the prevalence of HIV-1 dual infections in exclusively homosexual cohorts have been performed. It could be imagined that the frequency of dual infections will be different, as the transmission route for men having sex with men is dissimilar from heterosexual or IDUs, as is perhaps also the risk behaviour. Two large studies were performed on HIV-1 infected patients in Western Europe and the USA, with the majority being men having sex with men. A few larger studies have also been published to date. In 2003, the analysis of prot/RT sequences of 718 patients in San Francisco with at least two sampling moments and persistent viraemia during therapy showed that in none of these patients therapy failure was due to a HIV-1 superinfection [98]. In this study, degenerate base codes in the prot/RT sequence, representing viral mixtures, were artificially assigned a distance value to calculate nucleotide distances between serial samples. A large nucleotide distance between samples was taken as evidence for an HIV-1 superinfection. In addition, phylogenetic trees were constructed whereby monophyletic clustering of sequences from a single patient supposedly indicated evolution from a common ancestor, whereas paraphyletic clustering should imply HIV-1 superinfection. The HIV-1 prot/RT sequences routinely determined both at baseline and following therapy failure were used in a study in the Netherlands [73]. Patients were selected for further analysis based on the number of degenerate base codes in the RT fragment of this sequence. Additional sequence analysis confirmed that 16 of 37 (42%) patients had a dual HIV-1 infection, equalling 1% of the total 1661 records available. Another extensive survey of 660 HIV-1 seroconverters in France with samples collected from 1988–2004 did not discover any HIV coinfections or early superinfections in these patients using HMA as initial screening method [72]. In the studies from San Francisco, the Netherlands, and France, the risk group and the risk behaviour of the persons were not taken into account.

From the above studies, it cannot be concluded that the incidence of HIV-1 co- and superinfections is increasing. To reliably assess the incidence of HIV dual infections, additional cohort studies spanning an extended time of the epidemic are essential; these should encompass discrete risk groups of heterosexual, homosexual and shared needle transmission. However, it is clear that dual infections are more common in Africa than in the rest of the world, probably because of the significantly higher HIV-1 prevalence. A second HIV-1 infection occurs apparently as frequently as the first HIV-1 infection in African cohorts of heterosexual high risk individuals [33]. Frequent dual infection is reflected by the large number of recombinant viruses discovered in Africa, albeit such findings are not only restricted there [99].

From the studies reviewed above, it can be concluded that the main risk of HIV superinfection is high risk exposure, which consists of two components: HIV prevalence and risk behaviour. Another important factor is time since the initial infection, and the optimal time for a second infection is close to the initial infection. Viral determinants (fitness), host factors (immune response) and the mode of transmission seem to play less important roles.

A rise in the number of new HIV infections and other sexually transmitted diseases in men having sex with men has been reported since the introduction of HAART in the Western world, suggesting that an increase in HIV superinfections can also be expected in this risk group [100]. Men uninformed of their HIV status and seronegative men will likely also engage in more risky behaviour due to the availability of HAART treatment options. An acute infection can quickly be followed by HIV superinfection when most patients are still unaware of their HIV status. If superinfections usually take place close to the acute HIV infection, public counselling among HIV-infected individuals with known status is not likely to be effective in terms of prevention. Instead, overall public awareness campaigns of sexually transmitted disease prevention should be used to halt HIV superinfections with an emphasis on persons already infected with HIV to continue safe sex practices. Men having sex with men from San Francisco, who believed HIV superinfection can occur and that it damages their health, reported safer sex practices than men who did not believe in superinfection or did not believe that it could be harmful [101]. Serosorting (i.e. having unprotected sex only with persons of similar HIV serostatus) has been used as a HIV prevention strategy. However, serosorting may actually increase the chance of HIV transmission when partners are not aware of, or not sincere about, their HIV-1 status [102]. Unprotected sex with positive partners, which is becoming more and more frequent among HIV infected men having sex with men [102,103] is likely to boost the incidence of HIV superinfections, and should thus be discouraged.

## Conclusion

HIV-1 co-infection and superinfection are existing phenomena that contribute to viral diversity by the generation of recombinant viruses. The incidence of HIV superinfections is mainly controlled by risk exposure, which consists of two aspects: risk behaviour and HIV prevalence. Control by the immune system, in particular neutralizing antibodies, probably limits the time window of an HIV superinfection to the first two years after primary infection. In most, but not all superinfected patients, the second infection leads to faster disease progression. At present, it is unclear whether HIV-1 dual infections are increasing worldwide, but preliminary data from different cohorts suggest that dual infections increase when HIV-1 prevalence goes up, which is consistent with theoretical models.

## Competing interests

The author(s) declare that they have no competing interests.

## Authors' contributions

ACvdK and MC designed the review; ACvdK drafted the manuscript.
